# Robotic-assisted repair of colovesical anastomosis after Hartmann’s reversal procedure

**DOI:** 10.1590/S1677-5538.IBJU.2022.0453

**Published:** 2022-11-30

**Authors:** Jaime Poncel, Aref S. Sayegh, Oliver Ko, Rene Sotelo

**Affiliations:** 1 USC Institute of Urology Keck School of Medicine University of Southern California Los Angeles CA USA Catherine and Joseph Aresty, Department of Urology, USC Institute of Urology, Keck School of Medicine, University of Southern California, Los Angeles, CA, USA

## Abstract

**Purpose:**

Hartmann’s procedure is the resection of the rectosigmoid colon with an end colostomy formation and closure of the anorectal stump (1). Its reversal has a morbidity rate up to 58% (2, 3) with an incidence of fistulae formation of 4.08% (1). Herein, we present a robotic-assisted repair of a complex fistula that occurred as complication of Hartmann’s reversal when the stapler was introduced inadvertently through the vaginal canal.

**Patient and methods:**

Eighty-three-year-old female with past medical history of hysterectomy and ischemic colitis that required colectomy and colostomy placement in December 2020. In March 2022, the patient underwent a colostomy takedown, after which she reported fecaluria, urine leakage per vagina, and recurrent urinary tract infections. Cystoscopy and vaginoscopy revealed a large colovesical fistula, a staple in the bladder trigone, and several staples in the anterior vaginal wall. Robotically, extensive adhesiolysis was performed, the sigmoid was separated from the bladder, and the intact rectal stump was dissected free. The staple from the bladder trigone was removed. Bladder was closed in two layers with 3-0 V-Loc. Colorectal anastomosis was not feasible due to the short length of both ends. Therefore, a permanent colostomy was placed.

**Results:**

Operative time was 454min., and estimated blood loss was 100cc. Discharged on postoperative day 4 with a JP drain and a 20Fr Foley catheter. Drain, and Foley were removed on postoperative days 9 and 23, respectively. No postoperative complications were reported.

**Conclusion:**

Robotic-assisted repair represents an effective approach for the management of colovesical fistulae after Hartmann’s reversal.

**Figure 1:**
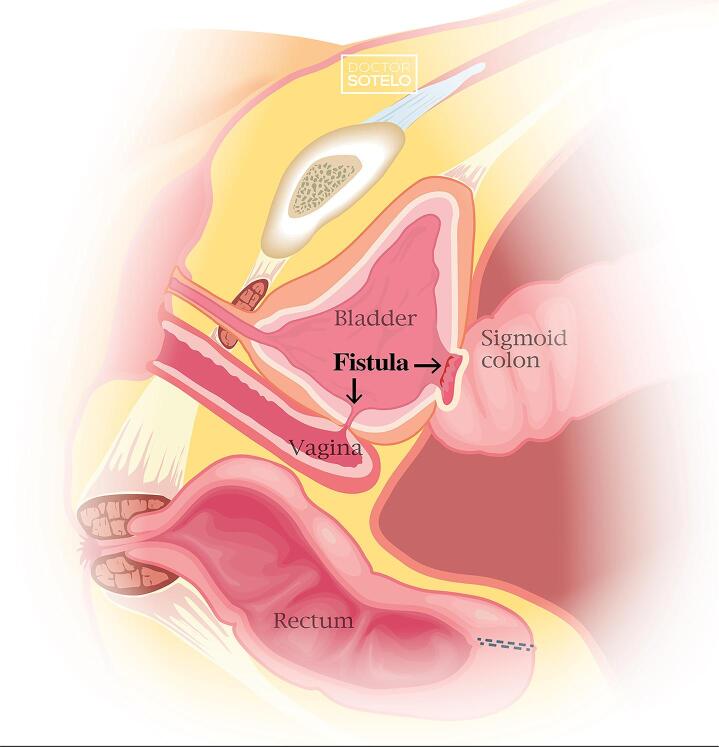
Colovesical and Vesicovaginal fistulae.
